# Automating pharmacovigilance evidence generation: using large language models to produce context-aware structured query language

**DOI:** 10.1093/jamiaopen/ooaf003

**Published:** 2025-02-08

**Authors:** Jeffery L Painter, Venkateswara Rao Chalamalasetti, Raymond Kassekert, Andrew Bate

**Affiliations:** GlaxoSmithKline, Durham, NC 27701, United States; GlaxoSmithKline, Durham, NC 27701, United States; Tech Mahindra, Plano, TX 75024, United States; GlaxoSmithKline, Philadelphia, PA 19104, United States; GlaxoSmithKline, London WC1A 1DG, United Kingdom; London School of Hygiene and Tropical Medicine, London WC1E 7HT, United Kingdom

**Keywords:** pharmacovigilance, drug safety, information retrieval, large language models (LLMs), natural language processing (NLP)

## Abstract

**Objective:**

To enhance the accuracy of information retrieval from pharmacovigilance (PV) databases by employing Large Language Models (LLMs) to convert natural language queries (NLQs) into Structured Query Language (SQL) queries, leveraging a business context document.

**Materials and Methods:**

We utilized OpenAI’s GPT-4 model within a retrieval-augmented generation (RAG) framework, enriched with a business context document, to transform NLQs into executable SQL queries. Each NLQ was presented to the LLM randomly and independently to prevent memorization. The study was conducted in 3 phases, varying query complexity, and assessing the LLM’s performance both with and without the business context document.

**Results:**

Our approach significantly improved NLQ-to-SQL accuracy, increasing from 8.3% with the database schema alone to 78.3% with the business context document. This enhancement was consistent across low, medium, and high complexity queries, indicating the critical role of contextual knowledge in query generation.

**Discussion:**

The integration of a business context document markedly improved the LLM’s ability to generate accurate SQL queries (ie, both executable and returning semantically appropriate results). Performance achieved a maximum of 85% when high complexity queries are excluded, suggesting promise for routine deployment.

**Conclusion:**

This study presents a novel approach to employing LLMs for safety data retrieval and analysis, demonstrating significant advancements in query generation accuracy. The methodology offers a framework applicable to various data-intensive domains, enhancing the accessibility of information retrieval for non-technical users.

## Introduction

Drug safety, or pharmacovigilance (PV), involves the systematic assessment of medications and vaccines to ensure that benefits outweigh risks. Central to PV are extensive databases compiling individual case safety reports (ICSRs), which are crucial for risk identification, strategy development, and regulatory reporting.^1^

Navigating safety databases to generate precise queries is inherently complex, requiring specialized expertise due to their vast, multi-table, and interlinked structures. Unlike many other relational databases, safety databases must manage diverse case types, regulatory requirements, and variations in product and event reports, all while ensuring compliance with stringent reporting standards. As noted by Brass and Goldberg, this complexity often arises from the mismatch between ontologies and relational databases,^2^ leaving users struggling to articulate accurate search criteria. The prevalence of query misapplication in healthcare further underscores the need for enhanced methods.^3^ Recent studies demonstrate the effectiveness of combining heuristic reasoning with deep learning for predicting semantic group assignments, achieving high accuracy and potentially supplementing automated query generation tasks.^4^ For example, the CHESS framework introduces a multi-component pipeline leveraging large language model (LLM)-based methods for entity and context retrieval, schema selection, and structured query language (SQL) generation, improving data retrieval in complex real-world databases.^5^

Our research presents a novel approach to converting natural language queries (NLQs) into SQL code for retrieving information from large, complex safety datasets. Safety databases present unique challenges, storing highly diverse data—ranging from case reports to product details, adverse events, and regulatory submissions—while complying with stringent reporting requirements across regions. Our company’s safety database contains over 500 tables, with more than 50 columns per table, spanning 50 years of data and encompassing at least 5000 distinct fields. The largest table holds nearly 1.3 billion rows, and a single safety case may include as many as 100 products and 100 events, adding to the complexity of tracking and querying. To address these challenges, we developed a *business context document* that distills intricate business rules and database knowledge from PV experts into accessible, plain language. Integrating this context document with the LLM enhances the model’s ability to craft queries that closely align with business needs, overcoming the limitations of relying solely on database metadata.

While research on NLQ-to-SQL tasks is ongoing, our approach significantly differs from frameworks like CHESS by introducing the business context document. This document enhances accuracy by providing the LLM with domain-specific knowledge, aligning query generation with business rules and database structures. Contextual knowledge is crucial for improving domain-specific NLQ tasks, with applicability beyond PV. Our work addresses the challenge of navigating an extremely large, complex database, a level of complexity not explicitly tackled by the CHESS framework.

Crafting queries in PV datasets requires both technical and scientific expertise, and translating safety scientists’ nuanced requests into precise code poses a significant challenge with considerable room for error.^6^ While tools like Query-by-Example (QBE) offer simplified means of crafting database queries,^7^ they fall short in handling complex needs.^8^

The rise of LLMs in natural language processing has improved data management by enabling more efficient and accurate query generation.^9–11^ However, LLMs alone often provide only reasonable, but not perfect, performance in domain-specific tasks.^12^ In heavily regulated environments like PV, accuracy is paramount, requiring approaches that integrate the expertise of domain specialists.

Recent evaluations highlight performance gaps between proprietary models like GPT-4 and open-source alternatives.^10^^,^^13^^,^^14^ While general query generation tasks have achieved up to 72% accuracy through advanced retrieval and schema selection techniques,^5^ LLM-generated SQL queries can suffer from issues like “hallucinations” and other errors.^15^ This underscores the need for supplementing LLMs with detailed contextual information to improve query precision in PV and other highly regulated domains.

Relying on technical teams for safety query formulation can introduce delays, underscoring the need for solutions that empower safety scientists with direct access to data through LLM-driven tools. Our approach builds on advancements in LLM technology, offering an intuitive interface that allows non-technical users to perform complex data queries, potentially enhancing PV data analysis and reporting.^12^ By integrating LLMs with a business context document, we aim to narrow the gap between technical complexity and domain expertise, enhancing access to critical drug safety informatics and supporting informed decision-making.

## Methods

Our study aimed to transform NLQs into Oracle™ SQL queries that are both executable code and semantically appropriate, utilizing LLMs within a retrieval-augmented generation (RAG) framework enhanced by a detailed business context document. For the purposes of this study, “executable code” refers to SQL queries that are free of structural errors and can be executed without modification, while “semantically appropriate” denotes queries that accurately fulfill the user’s intent by retrieving the correct data in response to the NLQ. The experiment was conducted in 3 phases to assess the LLM’s capability in SQL query generation under varying levels of contextual knowledge. The results of all NLQ-to-SQL generation was compared to a gold standard query generated by a subject matter expert (SME) to determine whether or not the intent was met by comparing the results of the generated code.

### Experimental phases

The experiment was structured into 3 phases, each targeting different aspects of LLM performance in translating NLQs into SQL. **Phase 1** established a baseline using an exhaustive database schema. **Phase 2** introduced a business context document, which provided plain language descriptions of data structures. **Phase 3** narrowed the focus to essential tables, aiming to determine whether a more targeted approach could enhance or match the performance observed in Phase 2.

**Figure 1.**
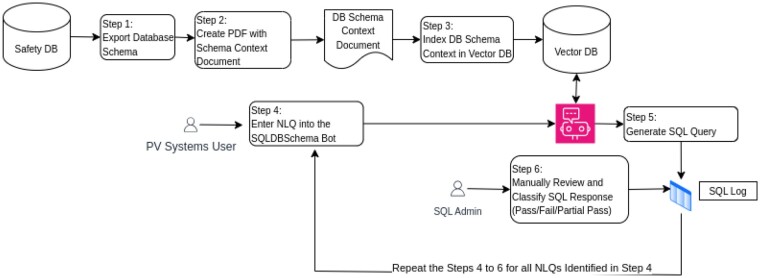
Phase 1 experimental design.

In **Phase 1**, as shown in Figure 1, the LLM was given comprehensive schema documentation, including a 290-page PDF detailing every table and column definition. This document was generated automatically by exporting the complete database schema using SQL Developer (https://www.oracle.com/database/sqldeveloper/). This file was then converted directly into a PDF for use by the RAG framework. It contains no additional content beyond the database definitions and metadata.

**Figure 2.**
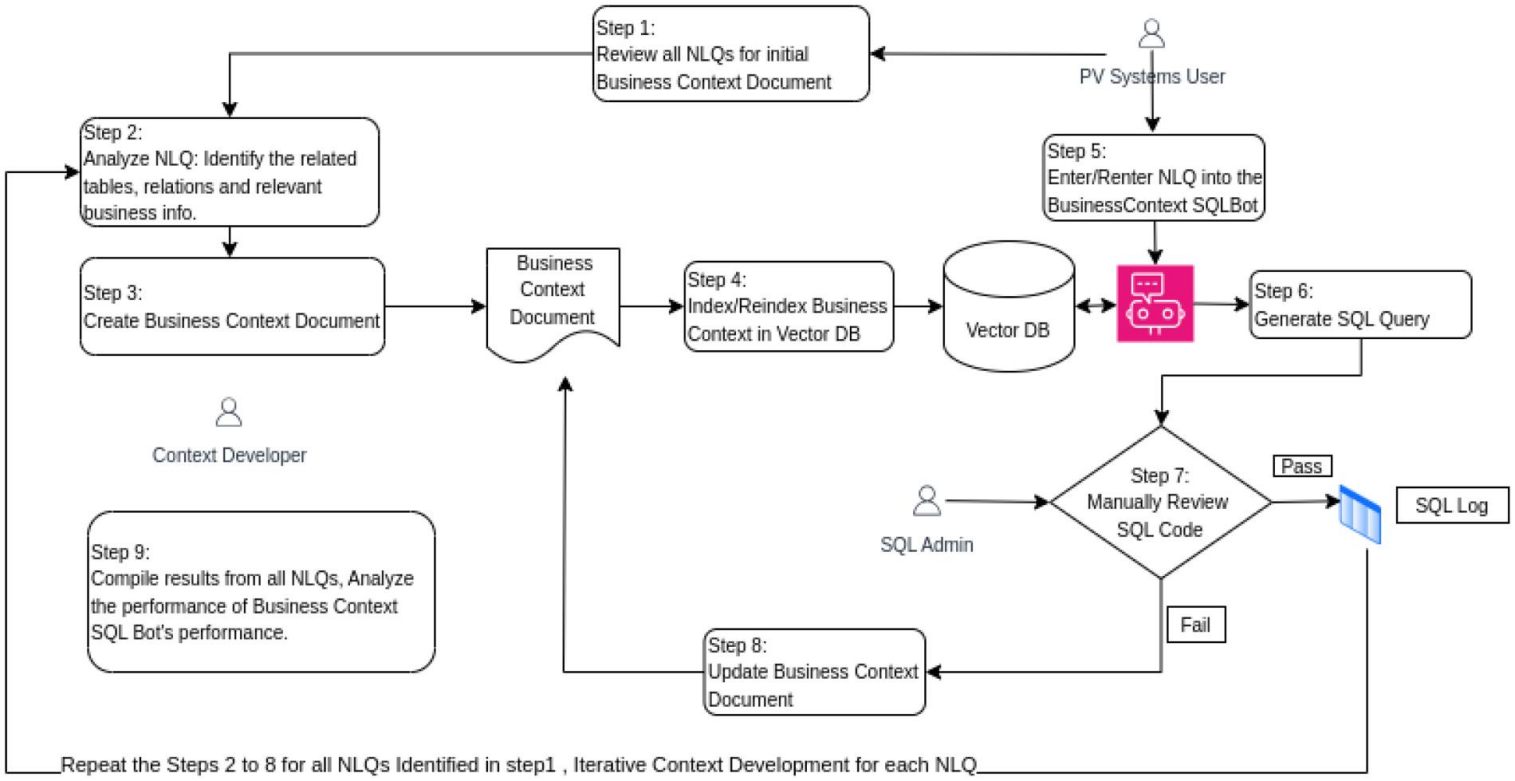
Phase 2 experimental design.

**Phase 2** mirrored Phase 1 but introduced a business context document, created by safety data experts. This document summarized key data structures in plain language, providing contextual insights into the relevance of the database elements (Figure 2). To promote transparency, we have prepared a redacted version of the business context document that includes only publicly available tables from vendor documentation. Refer to the Supplementary Files for insight into the structure and purpose of the document used in our study.

In Phase 3, the source document PDF created in Phase 1 was redacted to contain only 33 essential table definitions (see Appendix S1). These tables were selected based on our gold standard SQL queries, which provided a pre-defined benchmark for evaluating the accuracy of the LLM-generated queries across all phases of the experiment. From this set of pre-defined SQL queries, we identified the essential tables required to support successful query generation by the LLM. This approach ensured that the included corpus was comprehensive enough to answer all NLQs and gave the LLM the best possible opportunity to produce accurate SQL responses for this phase of the experiment. This streamlined approach focused the LLM on the most relevant data, aiming to improve query generation effectiveness. (The database used for storing our collected safety reports is a commercial product, so we cannot share the complete contents of the database schema. Readers interested in understanding the complexity of this system can refer to publicly available documentation from the software vendor. For more information, see the Oracle™ Argus documentation: https://docs.oracle.com/en/industries/life-sciences/argus-safety/8.4.3/index.html and the Argus Extensibility Guide: https://docs.oracle.com/health-sciences/argus-suite-82/argus-enterprise/AIEXG/AIEXG.pdf.)

### NLQ selection

Sixty NLQs were selected from historical user logs, covering a broad spectrum of query complexity (see Supplementary Data File [https://datadryad.org/stash/dataset/doi:10.5061/dryad.2280gb63n]). These queries were used consistently across all phases and ranged from simple to complex data retrieval tasks.

A structured prompt guided the LLM to generate syntactically correct SQL queries while minimizing unfounded responses. The chatbot prompt defined a specific persona, instructing the model:

“*You are an Oracle SQL expert. Given a question, generate a syntactically correct Oracle SQL query. Avoid querying non-existent columns and pay close attention to column-table associations. For keywords in the WHERE clause, ensure case-insensitive data comparison, for example, ‘upper(STATE_NAME) = upper(‘deleted’)’. If you are unable to generate the SQL query, please state that you cannot create the query without additional information or context, do not attempt to make anything up*.”

In all phases, a vector-based retrieval strategy, utilizing embeddings from the text-embedding-ada-002 model (https://platform.openai.com/docs/guides/embeddings/embedding-models), was employed. Each phase involved background knowledge tailored to the experiment—Phase 1 used the full schema, Phase 2 added contextual business knowledge, and Phase 3 focused on essential data elements.

**Figure 3.**
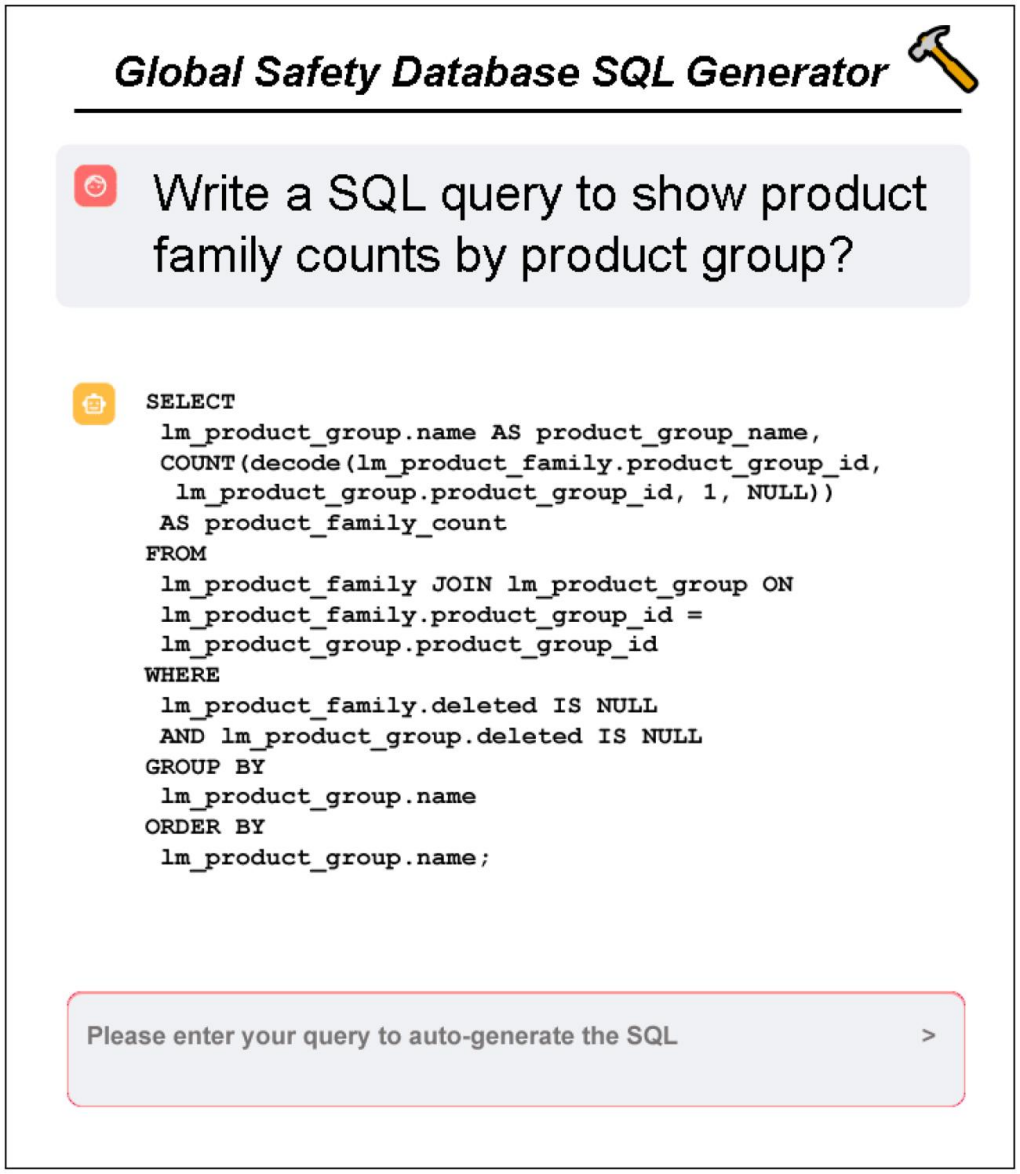
LLM Chatbot user interface.

Each experiment employed a text splitter (chunk size: 1000 characters; overlap: 200 characters) to process inputs for the GPT-4 model, as shown in Figure 3.

### Complexity scoring algorithm

For evaluating the performance of NLQ-to-SQL code generation, we developed a scoring algorithm to objectively measure SQL query complexity, which considers factors such as the number of tables, joins, and clauses in the query, as well as the estimated time required for manual creation. This method offers a reproducible way to evaluate SQL complexity, acknowledging that complexity assessments can vary widely in methodology,^16^ as shown in Algorithm 1. The complexity scores shared in our results were computed based on the gold standard SQL queries developed by our SME.

Algorithm 1. Compute the SQL Complexity Score

1: score←0

2: score←time_to_create

3: score←score+number_of_tables

4: score←score+number_of_joins

5: score←score+number_of_where_clauses

6: **if**  has_group_by=True  **then**

7:   score←score+1

8: **end if**

9: **if**  has_order=True  **then**

10:   score←score+1

11: **end if**

12: **if**  has_aggregation=True  **then**

13: score←score+1

14: **end if**

15: **return** *score*

### Experimental setup and classification of generated SQL

Each NLQ was presented to the LLM randomly and independently to prevent memorization. The context was reset between evaluations to ensure no data carried over between phases. A safety data expert evaluated each SQL query, categorizing them into *pass*, *fail*, or *partial pass* based on predefined criteria.

A “pass” was assigned to SQL queries that were executable without modifications and returned the same expected results as the manually created SQL query. This classification required the query to be syntactically correct, reference the correct table and column names, include logical JOIN conditions and WHERE clauses, and accurately capture the intent of the NLQ.

A “fail” occurred when the LLM could not generate a valid query. This included queries that were syntactically incorrect, referenced nonexistent tables or columns, or failed to capture the NLQ’s intent. Queries in this category either could not be executed or returned entirely incorrect results.

A “partial pass” was assigned to queries that were syntactically valid and executable but required minor modifications (no more than 2 edits) to produce correct results. For example, edits might correct a column reference or JOIN condition while maintaining the query’s logical intent. In addition, a query in this category may return data that was partially correct, such as including extraneous columns.

For all categories, a domain expert manually verified the generated SQL against a gold standard query to confirm its validity and ensure that results matched the expected output. This process removed any ambiguity in the classification criteria.

This classification approach informed the development of our experimental phases. Phases 1 and 2 evaluated performance with and without contextual knowledge, while Phase 3 focused the LLM on essential tables to assess whether this refinement improved performance.

The classification of results was consistent across all phases. For a query to qualify as a *pass* or *partial pass*, it had to (1) be fully executable, (2) retrieve the expected data, and (3) align with the intent of the NLQ. Queries in the *partial pass* category required only minor modifications, such as correcting table or column references, without changing the logical structure of the query. After these adjustments, *partial pass* queries returned results identical to the handcrafted SQL.

### Constructing the business context document

The business context document was developed for use in Phase 2 and served as a crucial differentiator in enhancing the LLM’s SQL generation capabilities. This document captured domain-specific knowledge in unstructured text form, prioritized frequently used tables aligned with regulatory and reporting requirements, and incorporated insights from SMEs. Unlike approaches such as CHESS, which may lack this depth of contextual understanding, our method ensured that the LLM had access to detailed domain knowledge to significantly improve query generation accuracy. The final business context document contained 33 essential table definitions, along with a glossary, business rules, and relationships necessary for query generation (see Appendix S1).

Examples of domain-specific guidance included:

**Definition** “*New case*”: *A new case includes cases not yet assigned to any database user (eg, Intake Specialist or Data Entry Specialist) as well as cases assigned but not yet accepted by the user.*

Additionally, explicit SQL generation suggestions were provided, such as:

- Avoid using SELECT * due to the vastness of some tables.
- *Always join tables using their primary and foreign keys.*
- *Consider database performance.*

In a production environment, maintenance of the business context document would be required, as updates to the database schema could impact the document’s utility, and reliability should be monitored over time. A redacted version of our business context document is provided as a supplement to the paper. The only portions redacted are proprietary data structures not publicly shared by the software vendor.

An illustrative example of the business context document in action is exemplified by one of the NLQs presented which requested a count of follow-up letters sent to patients. The database does not explicitly track letters, but the business context document specifies that these are stored in case attachment files, using the field CLASSIFICATION. For follow-up letters, the relevant SQL query rule is defined as CLASSIFICATION like ’%FU Attempt%’, bridging the gap between user queries and the database structure. Without our business context document, the LLM was unable to deduce this connection on its own from the raw schema.

### System design and implementation

LLMs demonstrate proficiency in answering general queries, but they often fall short when required to extract information from specific contexts without domain-specific training.^17^^,^^18^ Fine-tuning LLMs with contextual knowledge, such as business documents, significantly enhances their ability to generate accurate, contextually informed responses.^19^

To leverage this capability, we implemented a RAG-based pipeline tailored to SQL query generation in specific business contexts. While inspired by approaches described in prior work,^20^ our methodology diverges in key aspects to meet the unique requirements of our application. Specifically, we used LangChain to preprocess and split the business context document into manageable chunks, which were then embedded into vector representations using OpenAI’s text-embedding-ada-002 model. These embeddings were stored and indexed in a FAISS (Facebook AI Similarity Search) vector database, allowing for efficient similarity-based retrieval.

Our pipeline retrieves the top-7 most relevant document chunks for each user query using a similarity-based retriever interface. Unlike methods described in,^20^ which fine-tune retrievers and generators jointly, we rely on OpenAI’s GPT-4 (hosted on Azure) for the generation step without additional fine-tuning. The retrieved chunks serve as contextual input, enabling GPT-4 to generate accurate and syntactically correct SQL queries tailored to the schema and specific requirements outlined in the custom documentation.

For instance, if we asked the model to “generate a pivot table of product family counts by group,” the system would combine retrieved document chunks with query context and ensures compliance with database schema constraints (eg, matching columns to tables and handling case sensitivity in WHERE clauses). Importantly, during testing, we explicitly did not allow the system to retain conversational history between queries to ensure that each NLQ to SQL conversion was evaluated independently, avoiding any influence from prior interactions.

This hybrid approach—using pre-trained embeddings, vector-based retrieval, and generative LLMs—helps to improve SQL query generation contextually aligned with the business needs. The complete implementation details, including source code, are available in Appendix S1.

## Results

**Figure 4.**
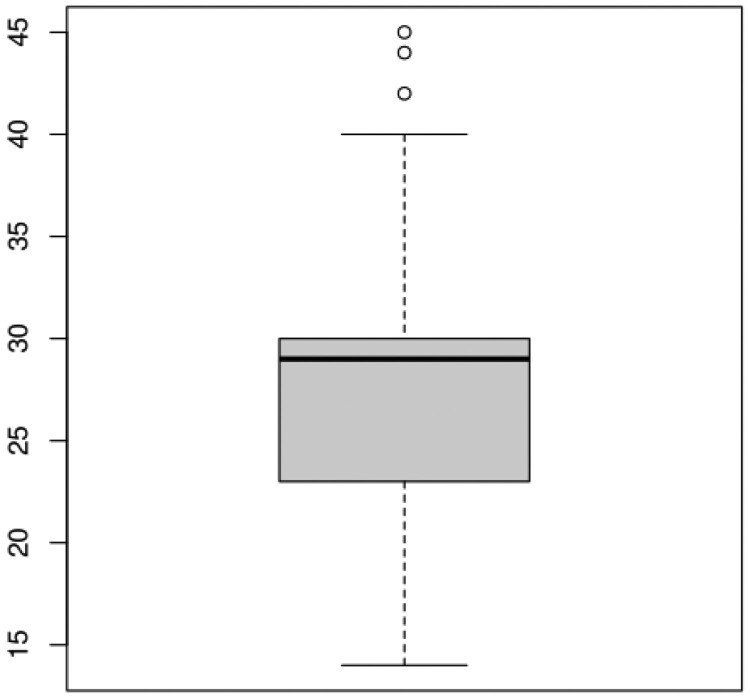
Boxplot of SQL complexity scores.

Our experiment evaluated the impact of integrating a business context document with LLMs on the complexity and accuracy of SQL queries generated from NLQs. After applying our scoring algorithm to the generated SQL queries, we conducted a distribution analysis of the complexity scores. Figure 4 illustrates this distribution, categorizing SQL query complexity as “low,” “medium,” or “high.” These categories were based on scoring percentiles: queries scoring below the 25th percentile were classified as “low,” those within the interquartile range (IQR) as “medium,” and those above the 75th percentile as “high.” This method provides an objective measure of SQL query complexity, incorporating both subjective expertise and quantifiable metrics like table count.

Of the 60 NLQs analyzed, 17 were categorized as “low” complexity, 31 as “medium,” and 12 as “high.” As outlined in the methods, the LLM-generated SQL queries were classified as “pass,” “partial success,” or “fail,” based on their accuracy. In Phase 1, where only the database schema was provided, the LLM achieved a pass rate of 8.3%, with 78.3% failing to generate valid SQL queries (Table 1). This highlighted the challenges of query generation without additional context.

**Table 1.** LLM performance with DB schema only.

Result	NLQ complexity				
	Low	Medium	High	Total	Percent
Pass	3	2	0	5	(8.3)
Fail	11	27	9	47	(78.3)
Partial pass	3	2	3	8	(13.3)
**Total**	17	31	12	60	

Abbreviations: DB = Database; LLM = Large Language Model; NLQ = Natural Language Query.

The integration of the business context document in Phase 2 significantly improved performance, increasing the pass rate to 78.3% (Table 2). This demonstrates the critical role of contextual knowledge in enhancing the LLM’s ability to generate accurate SQL queries, particularly for complex NLQs. Additionally, when excluding high-complexity queries, the LLM achieved an 85.4% pass rate, with 41 out of 48 low and medium complexity queries passing, and only 5 (10.4%) resulting in failure.

**Table 2.** LLM performance with business context document.

Result	NLQ complexity				
	Low	Medium	High	Total	Percent
Pass	16	25	6	47	(78.3)
Fail	0	5	0	5	(8.3)
Partial pass	1	1	6	8	(13.3)
**Total**	17	31	12	60	

Abbreviations: LLM = Large Language Model; NLQ = Natural Language Query.

Statistical analysis using Fisher’s Exact Test revealed a substantial improvement, with the *P*-value dropping from .1655 without the business context to .0006 with it, confirming the statistically significant performance boost provided by contextual information. Fisher’s Exact Test is a non-parametric statistical test commonly used when sample sizes are small and the data can be represented in a contingency table. This test was chosen for its robustness in handling categorical data and its suitability for our dataset, which involved discrete counts of query results classified as “pass,” “partial pass,” or “fail.” The *P*-value from this test quantifies the probability of observing the data (or something more extreme) under the null hypothesis that there is no difference between the groups being compared—in this case, the performance of the LLM with and without the business context document (see Appendix S1).

In our analysis:

- The *P*-value decreased from .1655 without the business context document to .0006 when the document was included, indicating a significant performance improvement.
- A *P*-value of .1655 suggests that the observed difference could occur by chance, meaning it is not statistically significant (typically, a *P*-value >.05 fails to reject the null hypothesis).
- A *P*-value of .0006, however, is well below the conventional threshold of .05, indicating that the observed improvement with the business context document is unlikely due to random chance. This confirms that the inclusion of contextual information significantly enhances the LLM’s performance in generating SQL queries.

The improvements were consistent across all complexity levels, highlighting the effectiveness of contextual priming in improving LLM SQL generation. Despite some remaining challenges with high-complexity queries, these results suggest that business context documents are a valuable tool for improving database interactions and LLM applications in data-intensive fields.

In **Phase 3**, the schema was narrowed to essential tables without including the business context document. This phase aimed to reduce ambiguity and assess baseline performance with a smaller set of relevant tables. The results showed modest improvements, reducing the failure rate from 78% to 50%. However, many queries still fell into the “partial pass” category, meaning they would require manual intervention to be revised into fully functional SQL queries (Table 3). A “partial pass” classification indicates that the query structure aligns with the intended outcome but needs minimal changes to be executable. Manual intervention typically involves correcting minor issues, such as typos or references to incorrect column names, while maintaining the overall structure that is efficient and appropriate for answering the user’s query. A query is classified as a “fail” if the LLM indicates it cannot generate a query, references the wrong tables, produces output inconsistent with the user’s NLQ request, or fundamentally answers the wrong question.

**Table 3.** Phase 3 LLM performance with narrowed schema definition.

Result	NLQ complexity				
	Low	Medium	High	Total	Percent
Pass	4	2	0	6	(10)
Fail	6	18	6	30	(50)
Partial pass	7	11	6	24	(40)
**Total**	17	31	12	60	

Abbreviations: LLM = Large Language Model; NLQ = Natural Language Query.

Fisher’s Exact Test for Phase 3 yielded a *P*-value of .2373, indicating that narrowing the schema led to modest improvements but did not match the success achieved with the business context document. These findings emphasize that while schema optimization can help, the comprehensive insights provided by the business context document play a more critical role in enhancing the LLM’s SQL query generation accuracy.

## Discussion

Through our multi-phase analysis, this study has shown the significant impact of context-enriched LLMs in enhancing data retrieval from NLQs within PV and other data-intensive domains. By integrating OpenAI’s GPT-4 model with a business context document, we markedly improved the model’s ability to generate syntactically precise and contextually relevant queries. This approach offers a promising pathway toward enhancing access to complex databases and enhancing the intuitiveness and efficiency of query formulation.

Our findings indicate that augmenting the LLM with contextual knowledge substantially improves query generation accuracy. Specifically, the introduction of a business context document resulted in a success rate exceeding 78% across a wide range of query complexities, highlighting the critical role of context in bridging the gap between natural language and database queries.

Recent literature has identified similar challenges in text-to-SQL generation. For example, Qu et al. identify common issues such as schema-based and logic-based hallucinations.^15^ Their Task Alignment (TA) strategy aims to mitigate these hallucinations by aligning tasks with familiar contexts. Our approach, which incorporates a business context document, appears to effectively eliminate many of these hallucinations by providing detailed, domain-specific knowledge that guides the LLM in generating accurate SQL queries. This alignment reduces the risk of generating erroneous or irrelevant queries.

Moreover, the CHESS framework’s benchmarking using the BIRD database presents an incongruent point of comparison. BIRD-SQL (https://bird-bench.github.io/) contains hundreds of sub-datasets; however, it does not clearly indicate the complexity of these sub-datasets, which may not reflect the challenges posed by enterprise databases like ours. Unlike the BIRD benchmarks, our complexity algorithm explicitly documents how the level of complexity was determined. Our enterprise database, which again contains over 500 tables with an average of more than 50 columns per table, encompasses multiple ambiguities and complex relationships. The database represents over 5 million safety cases, with the largest table containing nearly 1.3 billion rows, and a single safety case may have as many as 100 products and 100 events reported. These characteristics significantly impact the conversion of NLQs to valid SQL queries for retrieving relevant safety data. This suggests that the complexity of real-world applications is not adequately represented in BIRD-SQL, highlighting the need for more representative benchmarking datasets.

While our research seemingly marks a significant advancement in making informatics retrieval more accessible, enabling non-technical users to harness data-driven insights for more inclusive and efficient decision-making, it is essential to interpret our findings with caution. The efficacy of the business context document was assessed with a relatively small set of NLQs within a single enterprise database. The scalability and generalizability of our findings to other databases remain to be validated. Future research should aim to validate these results across broader datasets and diverse database architectures to fully understand the potential and limitations of our methodology.

Further advancements are necessary. Our system identifies key areas for additional research and development, particularly in handling complex queries and resolving ambiguous user intents. Concerns related to scalability and implementation within large, dynamic enterprise environments highlight the need for future investigations to enhance the robustness and applicability of our methodology.

## Conclusion

This study demonstrates the potential of leveraging LLMs, specifically OpenAI’s GPT-4, within a RAG framework to improve data retrieval from complex PV databases. Integrating a business context document significantly enhanced the model’s ability to generate accurate and contextually relevant SQL queries from NLQs, with the success rate increasing from 8.3% using the database schema alone to 78.3% with the context document.

Our findings emphasize the critical importance of contextual knowledge in bridging the gap between natural language and database queries, making data retrieval more intuitive and accessible for non-technical users. While these results are promising, further validation is needed to assess the scalability and generalizability of our approach across different databases and larger datasets.

Future research should aim to validate these findings across a variety of database architectures and more extensive datasets, exploring the methodology’s broader potential and limitations. Additionally, improving the system’s ability to handle complex queries and ambiguous user intents will be key to future development.

In summary, this study introduces a novel approach for employing LLMs in data retrieval through NLQs, improving the accessibility of PV data analysis. By integrating LLMs with a business context document, we propose a flexible pipeline that can be applied across multiple sectors, making complex databases more accessible to a wider range of users and supporting data-driven decision-making in various domains.

## Supplementary Data

Data available from the Dryad Digital Repository: https://doi.org/10.5061/dryad.2280gb63n.
